# Ice for Teething Children

**Published:** 1883-09

**Authors:** 


### ICE FOR TEETHING CHILDREN.

The pain of teething may be almost done away, and the health of the child benefited, by giving it fine splinters of ice, picked off with a pin to melt in its mouth. The fragment is so small that it is but a drop of warm water before it can be swallowed, and the child has all the coolness for its feverish gums without the slightest injury. The avidity with which the little things taste the cooling morsel, the instant quiet which succeeds hours of fretfulness, and the sleep which follows the relief, are the best witnesses to this magic remedy. Ice may be fed to a three-months' old child this way, each splinter being no larger than a common pin, for five or ten minutes, the result being that it has swallowed in that time a teaspoonful of warm water, which, so far from being a harm, is good for it, and the process may be repeated hourly as often as the fretting fits from teething begin.

---

House eaves should be guttered and spouted.

Swill-tubs should not be near doors or windows.

Outside channels should be in good order and be regularly cleaned.

Garden plats should, of course, be in order, and be properly not cultivated.

The ground floor of a house should not be below the level of the and, street or road outside.

The subsoil beneath a house should be naturally dry, or it should be made dry by land draining.

Cess-pools, cess-pits, sink-holes or drains should not be formed or retained within house basements.

The sub-soil within every basement should have a layer of concrete over it, and there should be full ventilation.

"If the various countries maintain the present rate of increase'," says Mr. Gosselin, Secretary of Embassy at Berlin, "fifty years hence the United States will have a population of 190,000,000, Russia approximately 153,000,000, Germany 83,000,000, the United Kingdom 63,000,000, Austria-Hungary and Italy both 44,000,000, France only 40,000,000.

"Father," said Johnnie, "this paper says that many prominent citizens are now ill with pneumonia and kindred diseases. What is a kindred disease, father?" "Why, my son," said Smithly, "a kindred disease is—is—why—yes, yes! a kindred disease is one that runs through an entire family—kindred, relatives, you know. Surprised you didn't know that, Johnnie."

